# Acute Conditioning of Antigen-Expanded CD8^+^ T Cells via the GSK3β-mTORC Axis Differentially Dictates Their Immediate and Distal Responses after Antigen Rechallenge

**DOI:** 10.3390/cancers12123766

**Published:** 2020-12-14

**Authors:** Pavla Taborska, Dmitry Stakheev, Hana Svobodova, Zuzana Strizova, Jirina Bartunkova, Daniel Smrz

**Affiliations:** Department of Immunology, Second Faculty of Medicine, Charles University and University Hospital Motol, 15006 Prague, Czech Republic; Pavla.Taborska@fnmotol.cz (P.T.); dmitry.stakheev@lfmotol.cuni.cz (D.S.); svobod.ha@seznam.cz (H.S.); Zuzana.Strizova@fnmotol.cz (Z.S.); jirina.bartunkova@lfmotol.cuni.cz (J.B.)

**Keywords:** CD8^+^ T cells, antigen rechallenge, cytokine starvation, Tim-3, mTOR, GSK-3β, TWS119, rapamycin, Torin1

## Abstract

**Simple Summary:**

Expanded, antigen-experienced CD8^+^ T cells are utilized in immunotherapy to treat infections and cancers. Antigen rechallenge of these cells leads to their re-expansion. The effector functions of re-expanded CD8^+^ T cells are critical for their therapeutic efficacy. We found that acute conditioning of the cells, before antigen rechallenge, impacts their effector function after re-expansion. Our data showed that acute pharmacological modulation of the GSK3β-mTORC axis with TWS119 or rapamycin, but not Torin1, before antigen rechallenge promotes the effector functions of re-expanded CD8^+^ T cells. These findings suggest that acute conditioning of the GSK3β-mTORC axis in expanded CD8^+^ T cells, before antigen rechallenge, can promote the therapeutic performance of re-expanded CD8^+^ T cells.

**Abstract:**

CD8^+^ T cells protect against tumors and intracellular pathogens. The inflammatory cytokines IL-2, IL-15, and IL-7 are necessary for their expansion. However, elevated serum levels of these cytokines are often associated with cancer, poorer prognosis of cancer patients, and exhaustion of antigen-expanded CD8^+^ T cells. The impact of acute conditioning of antigen-expanded CD8^+^ T cells with these cytokines is unknown. Here, we generated antigen-expanded CD8^+^ T cells using dendritic cells and PC-3 cells. The cells were acutely (18–24 h) conditioned with IL-2 and either the GSK3β inhibitor TWS119, the mTORC1 inhibitor rapamycin, or the mTORC1/2 inhibitor Torin1, then their immediate and post-re-expansion (distal) cytokine responses after antigen rechallenge were evaluated. We found that acute IL-2 conditioning upregulated the immediate antigen-induced cytokine response of the tested cells. Following their re-expansion, however, the cells showed a decreased cytokine response. These IL-2 conditioning-mediated impacts were counteracted with TWS119 or rapamycin but not with Torin1. Our data revealed that the acute conditioning of antigen-expanded CD8^+^ T cells with IL-2 modulates the GSK3β-mTORC signaling axis. This modulation differentially affected the immediate and distal cytokine responses of the cells. The acute targeting of this signaling axis could, therefore, represent a novel strategy for the modulation of antigen-expanded CD8^+^ T cells.

## 1. Introduction

CD8^+^ T cells are important players in the adaptive immune system [1]. Antigen challenge of naive CD8^+^ T cells with professional antigen-presenting cells induces their proliferation and differentiation into a variety of T cell subsets [1]. After antigen rechallenge, the proliferation of antigen-expanded CD8^+^ T cells resumes, and their effector functions become activated [2]. The most potent growth factors of naive and antigen-expanded CD8^+^ T cells are the inflammatory cytokines IL-2, IL-15, and IL-7 [3]. These cytokines can be elevated during cancer, chronic inflammatory infections, and immunotherapy [4,5,6,7,8,9]. High concentrations of these cytokines are also used during the ex vivo generation of T cells for adoptive cell transfer (ACT) [3,10]. Chronic exposure of antigen-expanded CD8^+^ T cells to these cytokines causes cell exhaustion [11]. The exhausted CD8^+^ T cells have decreased effector functions, and elevated proportions of exhausted CD8^+^ T cells are often associated with worse prognosis of the disease [11,12,13].

In addition to chronic exposure to these cytokines, antigen-expanded CD8^+^ T cells can also be exposed to acute stimulation with these cytokines. This situation can often occur during a cytokine storm elicited by an acute infection or cancer immunotherapy [14]. Whether this short, acute exposure to cytokines can simultaneously impact their immediate and post-re-expansion (distal) effector functions is unknown.

Multiple therapeutic approaches have been explored in an attempt to modulate cell signaling pathways and hence to modulate effector functions of CD8^+^ T cells [15,16,17,18,19]. Among these targetted signaling pathways are GSK-3β and the mammalian target of rapamycin (mTOR) [20,21,22], both of which can be regulated by the expression of the checkpoint molecule Tim-3 [23,24]. However, a large majority of these approaches rely on chronic interventions. Since the GSK-3β and mTORC signaling also regulate T cell proliferation and survival, the chronic interventions are limited and can produce side effects [25,26].

Acute interventions in cell signaling pathways have been shown to modulate cellular responses and/or activation phenotype [27,28,29]. These acute interventions can even produce opposing effects to those elicited by the chronic interventions [28]. However, as with the acute stimulation with cytokines, whether an acute modulation of CD8^+^ T cell signaling can simultaneously impact their immediate and post-re-expansion (distal) effector functions is unknown.

To investigate the impact of acute modulation of CD8^+^ T cell signaling on their immediate and post-re-expansion (distal) effector functions, we developed a novel and robust study system based on the use of allogeneic dendritic cells (DCs) and the prostate cancer cell line PC-3. This system allowed us to produce large numbers of antigen-expanded CD8^+^ T cells with antigen-independent and cytokine-mediated high and reversible expression of Tim-3 ex vivo. Using acute cytokine and pharmacological conditioning of the cells, we found that this conditioning differentially impacted their immediate and distal cytokine responses.

## 2. Results

### 2.1. Acute Starvation of IL-2 Diminishes the Robustness of the Immediate Response of Antigen-Expanded CD8^+^ T Cells after Rechallenge with Antigen

To investigate the impact of the inflammatory cytokine IL-2 on antigen-expanded CD8^+^ T cells, we developed a novel study model, based on the combined use of mature allogeneic monocyte-derived DCs (Supplementary Figure S1) and a PC-3 prostate cancer cell line (Supplementary Figure S2A). This system resulted in a greater than one hundredfold expansion of the initial number of lymphocytes (Supplementary Figure S2B) and their enrichment with CD4^+^ and CD8^+^ T cells (Supplementary Figure S2C).

**Figure 1:**
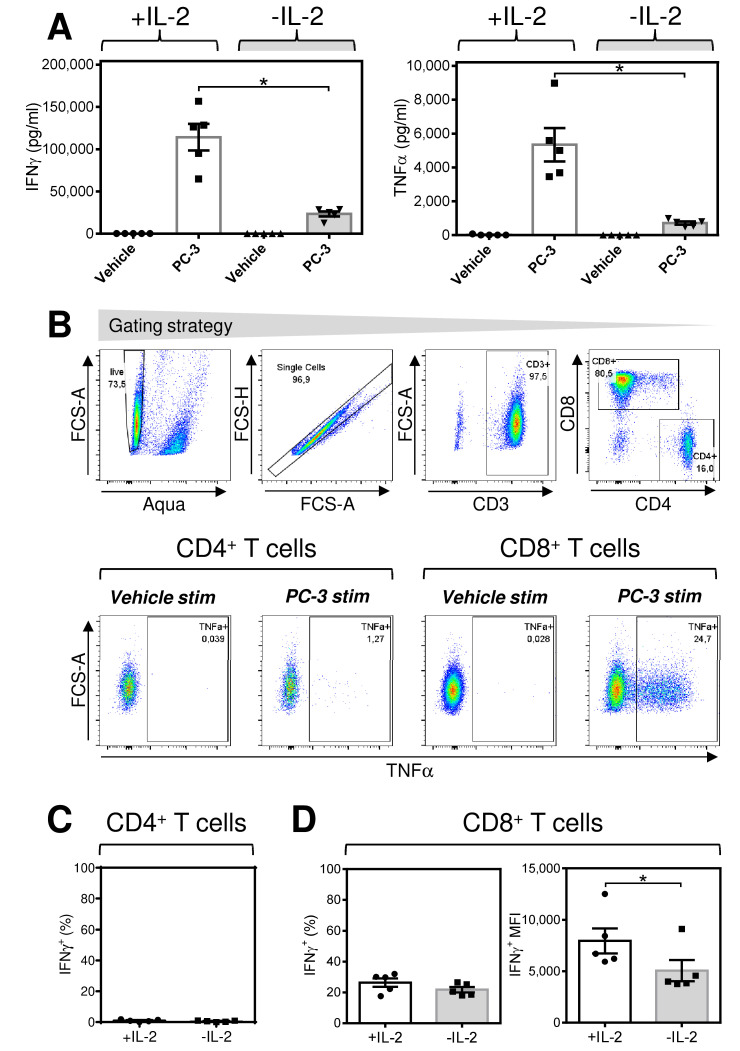

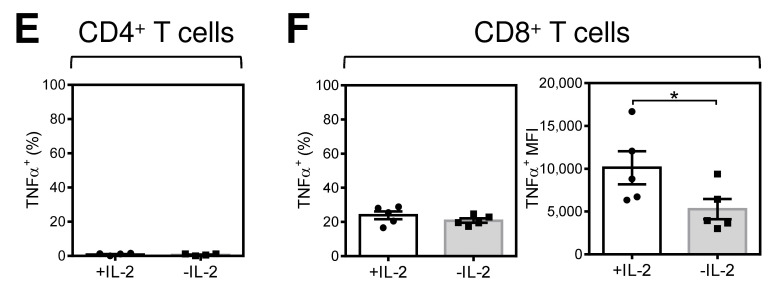
Acute starvation of IL-2 diminishes the robustness of the immediate response of expanded CD8^+^ T cells following stimulation with antigen. The 14-day-expanded lymphocytes were reconstituted and cultured for 18–24 h in LM medium supplemented with IL-2 (80 IU/mL). The cells were pelleted, washed two times with LM medium, and resuspended in fresh LM medium with or without IL-2 (80 IU/mL) and cultured for 18–24 h. The cells were washed, resuspended in fresh medium with or without IL-2 (80 IU/mL), and stimulated by coculturing with PC-3 cells at a ratio of 4:1 (lymphocytes:PC-3 cells). The released IFNγ and TNFα levels into the supernatant after 20-h stimulation were determined by ELISAs, and TNFα intracellular production after 5-h stimulation by flow cytometry. (**A**) The calculated concentrations (pg/mL) of IFNγ and TNF released into the supernatants by 1 × 10^6^/mL cell suspensions. (**B**) The gating strategy used to analyze the flow cytometry data. (**C**) The proportions of the IFNγ^+^ population of CD4^+^ T cells. (**D**) The proportion of the IFNγ^+^ population and its intensity of staining (MFI) of CD8^+^ T cells. (**E**) The proportions of the TNFα^+^ population of CD4^+^ T cells. (**F**) The proportion of the TNFα^+^ population and its intensity of staining (MFI) of CD8^+^ T cells. In (**A**,**C**–**F**), bars represent the mean of values determined in each group, and the significance of differences between the group of cells with (+) or without (−) IL-2 is indicated (* *p* < 0.05, *n* = 5 donors, paired 2-tailed Student’s *t* test).

We first investigated whether IL-2 in the culture might affect the immediate cytokine response of the generated antigen-expanded CD8^+^ T cells after antigen rechallenge. To do this, we compared the antigen-dependent production of the inflammatory cytokines IFNγ and TNFα in cells cultured for 18–24 h in the presence or absence of IL-2. Because PC-3 cells were used to provide the antigen to prepare the expanded T cells (Supplementary Figure S2A), we used them for stimulation. As shown in Figure 1A, stimulation of the expanded T cells by PC-3 cells induced a strong release of IFNγ and TNFα in the presence of IL-2. In the absence of IL-2, this release was significantly diminished (Figure 1A).

Intracellular cytokine analysis with flow cytometry showed that the source of these two inflammatory cytokines was CD8^+^ T cells because CD4^+^ T cells did not produce these cytokines after stimulation with PC-3 cells (Figure 1B,C,E). The analysis also revealed that the diminished cytokine release in the absence of IL-2 was not caused by a decrease in the PC-3 cell-reacting CD8^+^ T cell population (Figure 1D,F, left panels) but by a decline in the intensity of cytokine production, as shown by the staining intensity of IFNγ and TNFα (Figure 1D,F, right panels).

### 2.2. Acute Starvation of IL-2 before Antigen Rechallenge Promotes Re-Expansion of CD8^+^ T Cells with Enhanced Production of TNFα after Antigen Stimulation

**Figure 2:**
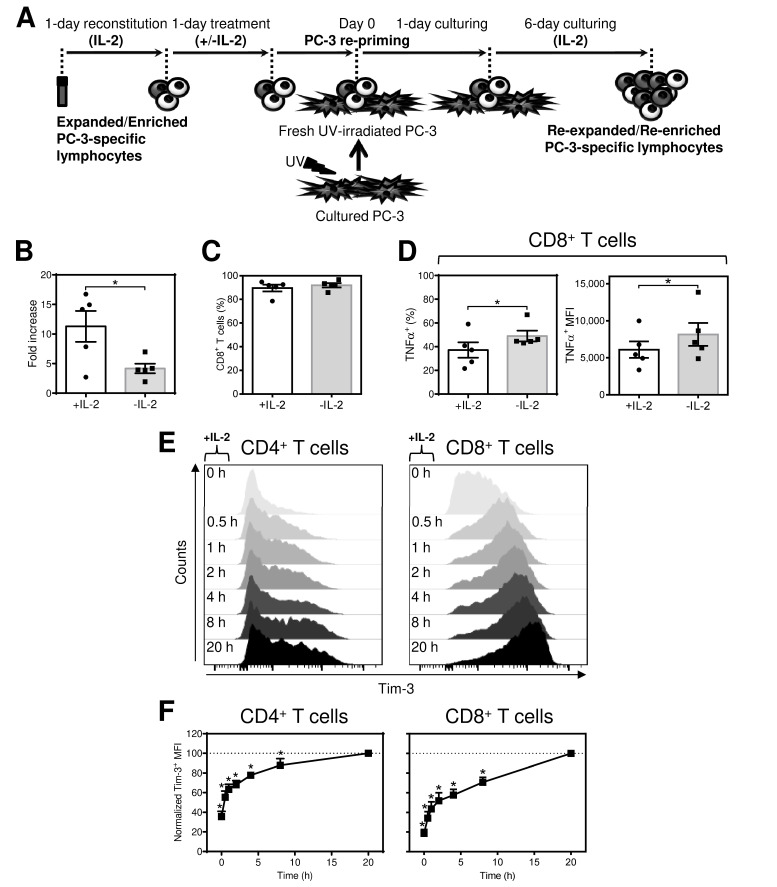
Acute starvation of IL-2 before antigen rechallenge promotes re-expansion of CD8^+^ T cells with enhanced production of TNFα after antigen stimulation. Acute IL-2 resupplementation enhances surface expression of Tim-3 in antigen-expanded T cells. The 14-day-expanded lymphocytes were reconstituted in the presence or absence of IL-2 and stimulated with UV-irradiated PC-3 cells as in Figure 1. After 18–24 h, the stimulated cells were resuspended and cultured in the presence of IL-2 (2000–4000 IU/mL) for 6 days. The re-expanded lymphocytes were counted, transferred to medium with fresh IL-2 (80 IU/mL) and stimulated with PC-3 cells as in Figure 1. The cells were analyzed by flow cytometry using the gating strategy in Figure 1B. (**A**) Schematic of lymphocyte re-expansion. (**B**) The cell number fold increase of the 7-day re-expanded lymphocytes. (**C**) The proportion of the CD8^+^ population of T cells in the re-expanded culture. (**D**) The proportion of the TNFα^+^ population (left panel) and its intensity of staining (MFI) (right panel) of CD8^+^ T cells. (**E**,**F**) The 14-day-expanded lymphocytes were reconstituted in the absence of IL-2. After 18–24 h, the starved cells were washed and resuspended in fresh LM medium with IL-2 (80 IU/mL). Intensities of Tim-3 staining (MFIs) of CD4^+^ T cells and CD8^+^ T cells after IL-2 resuplementation were determined at indicated time points (**E**) and evaluated (**F**). In (**B**–**D**), bars or data points represent the mean of values determined in each group, and the significance of differences between the group of cells with (+) or without (−) IL-2 is indicated (* *p* < 0.05, *n* = 5 donors, paired 2-tailed Student’s *t* test). In (**E**), data points represent the mean of values determined in each group, and the significance of differences between MFIs at 0 h and different time point is indicated (* *p* < 0.05, *n* = 3 donors, paired 2-tailed Student’s *t* test).

Although acute removal of IL-2 before antigen rechallenge inhibited the extent of the immediate response of antigen-expanded CD8^+^ T cells, we further investigated whether the removal also impacted the reactivity of re-expanded CD8^+^ T cells. To do this, we starved the expanded T cells of IL-2 and, still in the absence of IL-2, rechallenged them with PC-3 cells for 18–24 h (Figure 2A). The PC-3-rechallenged cells were then supplemented with a high concentration of IL-2 and cultured for 6 days (Figure 2A). The IL-2-starved T cells were found to expand significantly less than their nonstarved counterparts (Figure 2B). This response was associated with no significant change in the percentage of CD8^+^ T cells (Figure 2C). However, IL-2 starvation led to a significant enrichment of re-expanded CD8^+^ T cells with the population reacting to stimulation with PC-3 cells (Figure 2D, left panel). In addition, the response of this PC-3-reacting population was significantly enhanced compared with that of their nonstarved counterparts, as determined by the intensity of TNFα staining (Figure 2D, right panel). These data showed that although acute IL-2 starvation before antigen rechallenge of antigen-expanded CD8^+^ T cells led to a diminished immediate cytokine response, the subsequent expansion of rechallenged CD8^+^ T cells promoted enrichment of antigen-specific CD8^+^ T cells with an enhanced responsive phenotype.

### 2.3. Tim-3 Is a Reversible Marker of the Acute Impact of IL-2, IL-7, and IL-15 in Antigen-Expanded CD8^+^ T Cells

**Figure 3:**
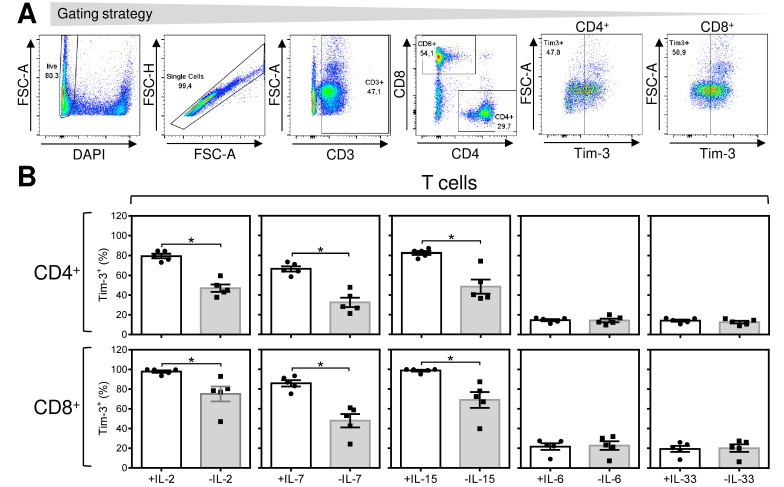

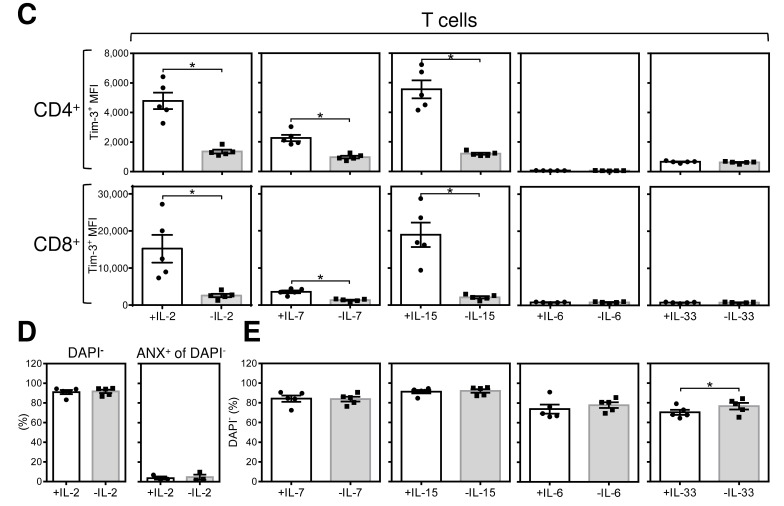
IL-2, IL-7, and IL-15 induce reversible surface expression of Tim-3 in antigen-expanded T cells. The 14-day-expanded lymphocytes were reconstituted and cultured for 18–24 h with IL-2 (133 IU/mL), IL-7 (13 IU/mL), IL-15 (133 IU/mL), IL-6 (666 IU/mL), or IL-33 (1333 IU/mL). The cells were washed and resuspended in fresh LM medium with or without cytokines. After 18–24 h, the cells were analyzed by flow cytometry for Tim-3 surface expression. (**A**) The gating strategy used to analyze flow cytometry data. (**B**) The proportions of the Tim-3^+^ population of CD4^+^ T cells and CD8^+^ T cells. (**C**) Intensities of Tim-3 staining (MFIs) of Tim-3^+^CD4^+^ T cells and Tim-3^+^CD8^+^ T cells. (**D**) The proportions of viable (DAPI^−^) cells and the proportions of their annexin V^+^ (ANX^+^) populations. The gating strategy for annexin V staining is shown in Figure S2D. (**E**) The proportions of viable (DAPI^−^) cells. In (**B**–**E**), bars represent the mean of values determined in each group, and the significance of differences between the group of cells with (+) or without (−) the indicated cytokine is indicated (* *p* < 0.05, *n* = 3 donors for annexin V staining data, *n* = 5 donors for other data, paired 2-tailed Student’s *t* test).

**Figure 4:**
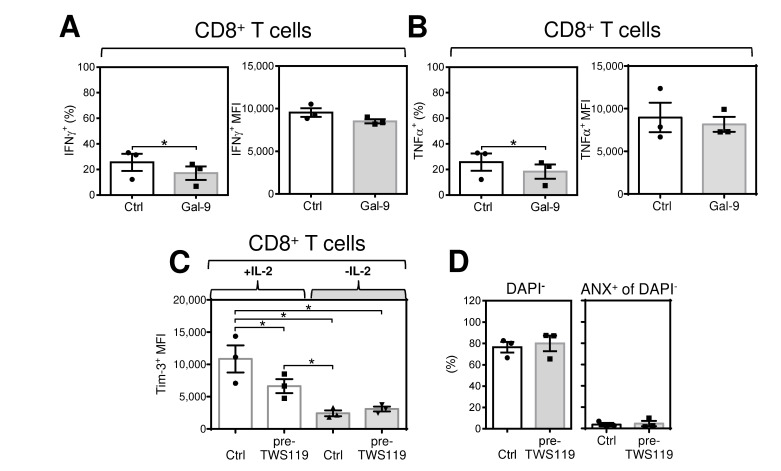

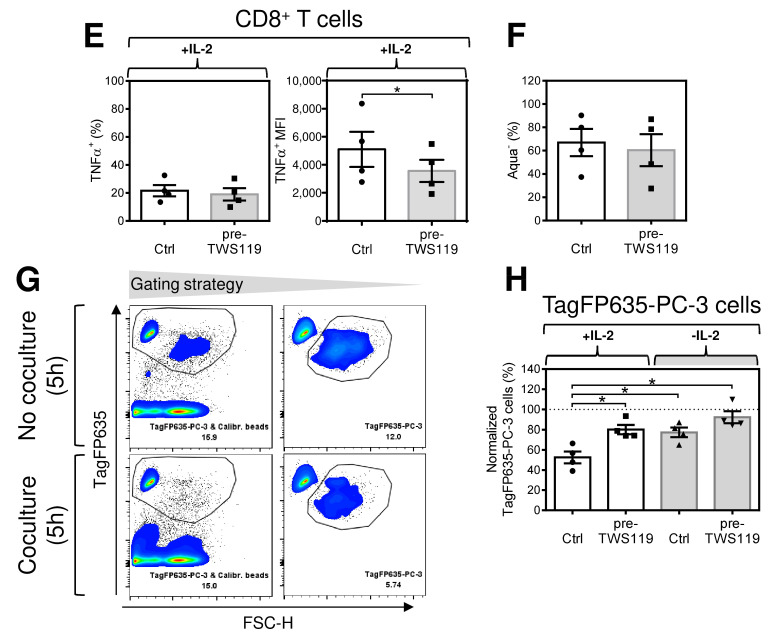
Galectin-9 inhibits the immediate response of expanded CD8^+^ T cells after stimulation with antigen. Acute conditioning of the expanded CD8^+^ T cells with the GSK-3β inhibitor TWS119 prior to antigen rechallenge decreases Tim-3 expression and diminishes the robustness of the immediate response of expanded CD8^+^ T cells after stimulation with antigen. (**A**,**B**) The impact of galectin-9 on expanded T cells. The 14-day-expanded lymphocytes were reconstituted in the presence of IL-2 as in Figure 1. The cells were (Gal-9), or were not (Ctrl), treated with recombinant galectin-9 (20 μg/mL) for 30 min, and then stimulated with PC-3 cells as in Figure 1. The cells were analyzed by flow cytometry using the gating strategy in Figure 1B. (**A**) The proportion of the IFNγ^+^ population and its intensity of staining (MFI) of CD8^+^ T cells. (**B**) The proportion of the TNFα^+^ population and its intensity of staining (MFI) of CD8^+^ T cells. (**C**,**D**) The impact of the GSK-3β inhibitor TWS119 on surface expression of Tim-3 and viability of expanded CD8^+^ T cells. The 14-day-expanded lymphocytes were reconstituted and cultured for 18–24 h with IL-2 (80 IU/mL). The cells were pelleted, resuspended in IL-2-containing (80 IU/mL), or not containing, medium supplemented with TWS119 (7 µM), and cultured for 18–24 h. (**C**) The intensity of Tim-3 staining (MFI) of expanded CD8^+^ T cells. (**D**) The proportions of viable (DAPI^−^) cells and the proportions of their annexin V^+^ populations. The gating strategy for annexin V staining is shown in Figure S2D. (**E**,**F**) The impact of the GSK-3β inhibitor TWS119 on the robustness of the immediate response of expanded CD8^+^ T cells after stimulation with antigen. The 14-day-expanded lymphocytes were reconstituted and treated as in (**C**,**D**) and then stimulated with PC-3 cells as in Figure 1. (**E**) The proportion of the TNFα^+^ population (left panel) and its intensity of staining (MFI) (right panel) of CD8^+^ T cells. (**F**) The proportion of viable (Aqua^−^) cells. (**G**,**H**) The cytotoxic impact of the 14-day-expanded lymphocytes on fluorescent TagFP635-PC-3 cells. The 14-day-expanded lymphocytes were reconstituted and treated as in (**C**,**D**) and then stimulated (cocultured) with TagFP635-PC-3 cells as in Figure 1. The proportions of TagFP635-PC-3 cells in the cultures were determined by flow cytometry. (**G**) The gating strategy used to analyze flow cytometry data. (**H**) Normalized proportions of TagFP635-PC-3 cells after coculture with lymphocytes. The proportions were normalized to TagFP635-PC-3 cells not cocultured with lymphocytes (100 %). In (**A**–**H**), bars represent the mean of values determined in each group. In (**A**–**C**) (left panel), and (**D**–**F**), the significance of differences between the group of cells is indicated (* *p* < 0.05, (**A**,**B**,**D**): *n* = 3 donors, (**C** (left panel) and **E**,**F**): *n* = 4 donors, paired 2-tailed Student’s *t* test). In (**C**) (right panel) and (**H**), the significance of differences among the group of cells is indicated (* *p* < 0.05, (**C**) (right panel): *n* = 3 donors, (**H**): *n* = 4 donors, 1-way ANOVA with the Tukey post-test).

To further study the acute and reversible antigen-independent impact of IL-2 on antigen-expanded CD8^+^ T cells, we searched for a marker that would reflect this event. One of the molecules whose expression is induced by IL-2 and other common γ-chain cytokines is Tim-3 [30]. We first confirmed that IL-2 induced a surface expression of Tim-3 in IL-2-starved, antigen-expanded CD4^+^ and CD8^+^ T cells (Figure 2E,F). In subsequent experiments, we compared Tim-3 expression in expanded cells cultured overnight (18–24 h) in the presence or absence of cytokines (starvation). In the presence of IL-2, Tim-3 was highly expressed in the majority of both CD4^+^ and CD8^+^ T cells (Figure 3B, left panels). However, overnight removal of IL-2 from the culture led to a significant decrease in the proportion of Tim-3-expressing cells (Figure 3B, left panels). The impact on Tim-3 expression was even more profound for the Tim-3 staining intensity (Figure 3C left panels). Similar results were obtained for the cytokines IL-7 and IL-15 (Figure 3B,C, second and third left panels). Minimal expression of Tim-3 was, however, observed with the cytokines IL-6 and IL-33 (Figure 3B,C, two right panels). The downregulated Tim-3 expression was found to be restored after overnight resupplementation of the cultures with pertinent γ-chain cytokines (data not shown). Regardless of the changes in Tim-3 expression, cytokine starvation was not associated with a decrease in cell viability as determined by annexin V and/or DAPI staining (Figure 3D,E). These data showed that high levels of Tim-3 expression are maintained by the acute presence of the tested γ-chain inflammatory cytokines in the expanded T cells and that this expression is reversed or restored after their removal or resupplementation, respectively. In addition, a Tim-3 ligand, galectin-9 [31,32], inhibited the inflammatory response of antigen-expanded CD8^+^ T cells, supporting an inhibitory function of Tim-3 in these cells (Figure 4A,B).

### 2.4. Acute Conditioning of Antigen-Expanded CD8^+^ T Cells with the GSK-3β Inhibitor, TWS119, Prior to Antigen Rechallenge Decreases IL-2-Mediated Tim-3 Expression and the Robustness of Their Immediate Response to Antigen Rechallenge

In the next series of experiments, we used Tim-3 as a marker of the acute impact of IL-2 on expanded CD8^+^ T cells and searched for cell signaling inhibitors that would acutely decrease the expression of Tim-3 in these cells in the presence of IL-2.

IL-2-mediated stimulation employs the mTOR signaling axis [33,34,35]. One of the mTOR regulators is GSK-3β, which regulates mTOR complex 1 (mTORC1) [36] and was previously reported to enhance CD8^+^ T cell effector functions [37]. Tim-3 expression was also found to regulate GSK-3β signaling [24]. Therefore, we tested whether pharmacologically-induced inhibition of GSK-3β with TWS119 [38] can counteract IL-2-mediated Tim-3 expression and acute enhancement of the CD8^+^ T cell responsive phenotype. Using β-catenin translocation as a surrogate marker of GSK-3β activity [15], we first confirmed by imaging flow cytometry [39] that TWS119 inhibited the GSK-3β activity in the antigen expanded CD8^+^ T cells (Figure S2E). In the following experiments, we found that the acute conditioning of expanded T cells with TWS119, in the presence of IL-2, significantly decreased the Tim-3 staining intensity (Figure 4C). In addition, TWS119 in the IL-2-starved CD8^+^ T cells did not further decrease the Tim-3 staining intensity, indicating that IL-2 starvation and TWS119 downregulated the Tim-3 expression in the cells via the same mechanism. Importantly, although TWS119-mediated acute conditioning did not decrease the proportion of TNF-producing CD8^+^ T cells (Figure 4E, left panel), the TNFα staining intensity of the reacting CD8^+^ T cells was decreased (Figure 4E, right panel). In addition, no impact of the conditioning on the viability of the tested cells was observed (Figure 4D,F).

Next, we tested whether IL-2-starvation- or TWS119-elicited inhibition of the immediate inflammatory response of antigen-expanded CD8^+^ T cells also inhibited the cytotoxic activity of antigen-expanded cells after antigen rechallenge. Using fluorescent TagFP635-PC-3 cells [39] as both the target cells and source of the antigen, we found that both IL-2-starvation and TWS119-mediated acute conditioning decreased the cytotoxic activity of antigen-expanded cells (Figure 4G,H). These data showed that acute conditioning with TWS119 could mimic IL-2 starvation by affecting the expression of Tim-3 and the acute responsive phenotype in antigen-expanded CD8^+^ T cells.

### 2.5. Acute Conditioning of Antigen-Expanded CD8^+^ T Cells with the GSK-3β Inhibitor TWS119 Prior to Antigen Rechallenge Promotes Re-Expansion of CD8^+^ T Cells with Enhanced Production of TNFα after Antigen Stimulation

**Figure 5:**
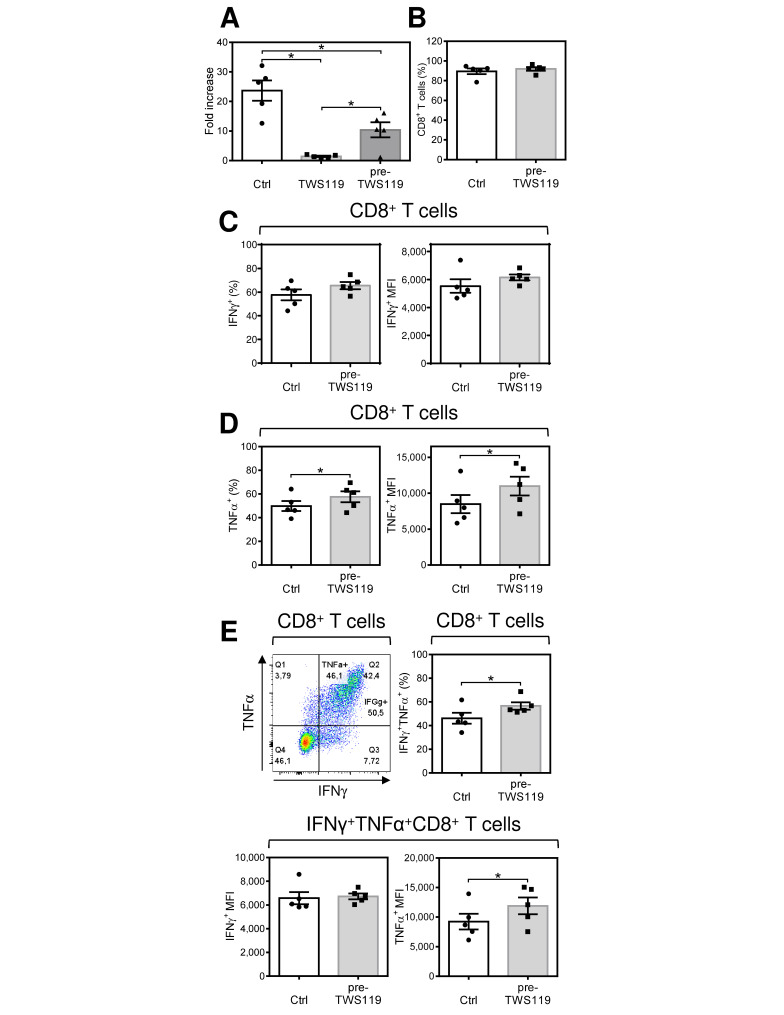
Acute conditioning of the expanded CD8^+^ T cells with the GSK-3β inhibitor TWS119 prior to antigen rechallenge promotes re-expansion of CD8^+^ T cells with enhanced production of TNFα after antigen stimulation. The 14-day-expanded lymphocytes were reconstituted and cultured for 18–24 h with IL-2 (80 IU/mL). The cells were pelleted, resuspended in IL-2-containing medium (80 IU/mL) supplemented with TWS119 (7 µM), and cultured for 18–24 h. The cells were pelleted, resuspended in fresh IL-2-containing medium (80 IU/mL), stimulated with UV-irradiated PC-3 cells as in Figure 1, and 7-day re-expanded and analyzed as in Figure 2. (**A**) The cell number fold increase of the 7-day re-expanded lymphocytes with TWS119 during pretreatment and expansion (TWS119), during pretreatment (pre-TWS119), or without TWS119 (Ctrl). (**B**) The proportion of the CD8^+^ population of T cells in the re-expanded culture. (**C**) The proportion of the IFNγ^+^ population (left panel) and its intensity of staining (MFI) (right panel) of CD8^+^ T cells. (**D**) The proportion of the TNFα^+^ population (left panel) and its intensity of staining (MFI) (right panel) of CD8^+^ T cells. (**E**) The gating strategy for the IFNγ^+^TNFα^+^ population of CD8^+^ T cells (top left panel), the proportion of the IFNγ^+^TNFγ^+^ population of CD8^+^ T cells (top right panel) and the IFNγ^+^(bottom left panel) and TNFα^+^ (bottom right panel) intensities of staining (MFI) of IFNγ^+^TNFα^+^ population of CD8^+^ T cells. In (**A**), bars represent the mean of values determined in each group, and the significance of differences among the group of cells with TWS119 during pretreatment and expansion (TWS119), during pretreatment (pre-TWS119), or without TWS119 (Ctrl) is indicated (* *p* < 0.05, *n* = 5 donors, 1-way ANOVA with Tukey’s posttest). In (**B**–**E**), bars represent the mean of values determined in each group, and the significance of differences between the groups is indicated (* *p* < 0.05, *n* = 5 donors, paired 2-tailed Student’s *t* test).

Next, we tested whether pharmacologically-induced inhibition of GSK-3β with TWS119 can also mimic IL-2 starvation by impacting the responsive phenotype revealed in the re-expanded CD8^+^ T cells. In the first set of experiments, we pretreated (acutely conditioned), PC-3-stimulated, and then re-expanded CD8^+^ T cells in the presence of IL-2 with the GSK3β inhibitor TWS119. We found that the inhibitor completely abrogated the re-expansion of CD8^+^ T cells (Figure 5A). Therefore, we used the inhibitor only for T cell pretreatment prior to antigen rechallenge. Under this condition, the cells substantially regained their expansion capacity (Figure 5A) with no impact on the proportion of CD8^+^ T cells (Figure 5B). We next analyzed the activation phenotype. As shown in Figure 5C, the pretreatment of CD8^+^ T cells with TWS119 prior to antigen rechallenge had no impact on the proportion of antigen-specific IFNγ-producing CD8^+^ T cells (Figure 5C, left panel) or the extent of IFNγ production as determined from the intensity of the intracellular cytokine staining (Figure 5C, right panel). However, TWS119 pretreatment increased the proportion of antigen-specific TNFα-producing CD8^+^ T cells (Figure 5D, left panel) and the intensity of TNFα production (Figure 5D, right panel). Comparable data were obtained for the antigen-specific IFNγ/TNFα-producing CD8^+^ T cell population because TWS119 increased its proportion in the expanded CD8^+^ T cells (Figure 5E, top right panel). However, it was only the production of TNFα (Figure 5E, bottom right panel) but not IFNγ (Figure 5E, bottom left panel) that was increased as determined from the cytokine staining intensities of the stimulated cells (Figure 5E, bottom panels). These data showed that acute pretreatment/conditioning of antigen-expanded CD8^+^ T cells with TWS119 could impact their activation phenotype after antigen rechallenge and re-expansion.

### 2.6. Acute Conditioning of Expanded CD8^+^ T Cells with Rapamycin, but Not with Torin1, Prior to Antigen Rechallenge Promotes Re-Expansion of CD8^+^ T Cells with Enhanced Production of TNFα and IFNγ after Antigen Stimulation

GSK3β is known to be a regulator of mTOR signaling complexes, mTOR complex 1 (mTORC1) and mTOR complex 2 (mTORC2) [40]. In addition, Tim-3 expression was found to regulate mTORC signaling [23]. To test whether acute conditioning of CD8^+^ T cells, through the modulation of GSK3β-regulated signaling targets, can also impact the effector function of antigen-rechallenged and re-expanded CD8^+^ T cells, we used two inhibitors of mTORCs: rapamycin, an mTORC1 inhibitor [41], and Torin1, an inhibitor of both mTORC1 and mTORC2 [42].

**Figure 6:**
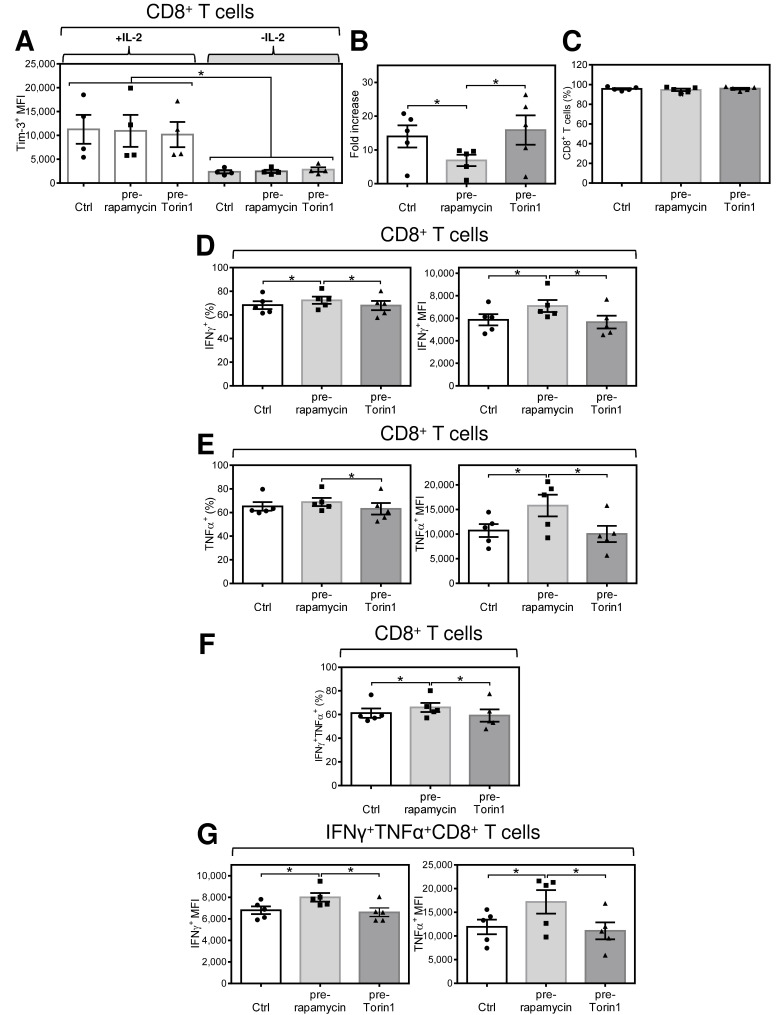
Acute conditioning of expanded CD8^+^ T cells with the mTOR complex inhibitor rapamycin but not Torin1 prior to antigen rechallenge promotes re-expansion of CD8^+^ T cells with enhanced production of TNFα and IFNγ after antigen stimulation. (**A**) The intensity of Tim-3 staining (MFI) of expanded CD8^+^ T cells. The 14-day-expanded lymphocytes were reconstituted and cultured for 18–24 h with IL-2 (80 IU/mL). The cells were pelleted, resuspended in IL-2-containing medium (80 IU/mL) supplemented with 100 nM rapamycin or 100 nM Torin1, and cultured for 18–24 h. The cells were pelleted, resuspended in fresh IL-2-containing medium (80 IU/mL), stimulated with UV-irradiated PC-3 cells as in Figure 1, and 7-day re-expanded and analyzed as in Figure 2. (**A**) The intensity of Tim-3 staining (MFI) of expanded CD8^+^ T cells after their acute conditioning with rapamycin (pre-rapamycin) and Torin1 (pre-Torin1) prior to antigen rechallenge and re-expansion. (**B**) The cell number fold increase of the 7-day re-expanded lymphocytes not pretreated (Ctrl) or pretreated with rapamycin (pre-rapamycin) or Torin1 (pre-Torin1). (**C**) The proportion of the CD8^+^ population of T cells in the re-expanded culture. (**D**) The proportion of the IFNγ^+^ population (left panel) and its intensity of staining (MFI) (right panel) of CD8^+^ T cells. (**E**) The proportion of the TNFα^+^ population (left panel) and its intensity of staining (MFI) (right panel) of CD8^+^ T cells. (**F**) The proportion of the IFNγ^+^TNFα^+^ population of CD8^+^ T cells. (**G**) The IFNγ^+^ (left panel) and TNFα^+^ (right panel) intensities of staining (MFI) of IFNγ^+^TNFα^+^ population of CD8^+^ T cells. In (**A**–**G**), bars represent the mean of values determined in each group, and the significance of differences among the tested groups is indicated (* *p* < 0.05, *n* = 5 donors, 1-way ANOVA with the Tukey post-test).

We first confirmed that both rapamycin and Torin1 efficiently inhibited mTORC signaling in antigen-expanded lymphocytes as determined by the inhibition of Ser235|236 phosphorylation in ribosomal protein S6 (Supplementary Figure S2F,G) [43,44]. In the following experiments, we found that, regardless of the presence or absence of IL-2, acute inhibitor treatment of expanded CD8^+^ T cells did not decrease their Tim-3 surface expression (Figure 6A). These data showed that IL-2 and GSK-3β activity upregulate Tim-3 surface expression upstream of mTORCs and that mTORCs do not upregulate the acute and reversible IL-2-induced surface expression of Tim-3 in antigen-expanded CD8^+^ T cells.

In the subsequent analyses, we found that pretreatment of CD8^+^ T cells with Torin1 prior to antigen rechallenge had no impact on the subsequent cell expansion (Figure 6B). No effect was observed on the proportion of antigen-specific CD8^+^ T cells (Figure 6C) or their inflammatory response (Figure 6E,G). Pretreatment with rapamycin also did not alter the proportion of re-expanded CD8^+^ T cells in culture (Figure 6C). However, rapamycin decreased the expansion of the cells in the culture by approximately 50% (Figure 6B). Rapamycin slightly enriched the cell culture with the antigen-specific CD8^+^ T cell population (Figure 6D, left panel). A notable impact of rapamycin was, however, shown on the extent of IFNγ and TNFα production. As shown in Figure 6D,E (right panel), the intensity of the intracellular cytokine staining in the antigen-reacting CD8^+^ T cell populations was significantly increased in the rapamycin-pretreated cells. Rapamycin pretreatment also slightly enhanced the proportion of the antigen-specific IFNγ/TNFα-producing CD8^+^ T cell population (Figure 6F). This population also produced significantly more of these inflammatory cytokines, as determined from the intracellular cytokine staining intensities (Figure 6G). Overall, rapamycin-mediated acute conditioning of CD8^+^ T cells prior to antigen rechallenge had a similar impact on re-expanded cells as conditioning with the GSK-3β inhibitor TWS119. However, in contrast to TWS119, rapamycin did not impact the surface expression of Tim-3 in these cells.

## 3. Discussion

This study describes a novel mode of regulation of the effector functions of antigen-expanded CD8^+^ T cells. Using a novel model system, we showed that acute conditioning of expanded CD8^+^ T cells with IL-2, before antigen rechallenge, produces a much more robust immediate inflammatory response than acute conditioning via IL-2 starvation. However, the opposite effect of IL-2-mediated acute conditioning was found in the distal responses after cell re-expansion. Using Tim-3 as a reversible marker of IL-2-mediated stimulation of antigen-expanded CD8^+^ T cells, we identified GSK3β-mTORC signaling as the acute regulator of the immediate and distal responsive phenotypes of antigen-expanded CD8^+^ T cells.

Antigen-expanded CD8^+^ T cells are important for protection against disease recurrence. Upon antigen rechallenge, the cells can rapidly re-expand and, hence, provide this protection [45]. Many studies have shown that the conditioning of these cells affects their activation phenotype [46]. Changes in the activation phenotype are often a result of chronic stimulation, occurring upon prolonged infections or in the tumor microenvironment [47]. This chronic stimulation can cause irreversible changes in the cell responsive phenotype, often leading to T cell exhaustion [48]. One of the markers of T cell exhaustion is Tim-3 [49]. Tim-3 is associated with a dysfunctional phenotype of CD8^+^ T cells in chronic infectious diseases as well as in multiple cancers [11]. However, Tim-3 expression is also a marker of antigen-independent stimulation of T cells by inflammatory γ-chain cytokines, such as IL-2, IL-7, and IL-15 [30]. Our data confirmed that these cytokines induce the expression of Tim-3 in antigen-expanded CD8^+^ T cells. However, our data also showed that this cytokine-induced expression of Tim-3 is highly reversible because removing the cytokines from the culture media reversed Tim-3 expression in the expanded CD8^+^ T cells. We observed that the extent of Tim-3 expression was also dependent on the cytokine concentration in the media (data not shown). The data indicated that elevated concentrations of inflammatory cytokines under disease conditions [4,5,6,50] can reversibly upregulate Tim-3 expression in circulating or tissue-infiltrating expanded CD8^+^ T cells. If so, Tim-3 expression in these cells should not necessarily indicate their exhaustion but is also a reflection of the actual inflammatory burden with γ-chain cytokines in the place of the T cell residence. Moreover, our data suggest that Tim-3 surface expression in antigen-expanded CD8^+^ T cells could be downregulated via the inhibition of GSK-3β. This is similarly to what has been reported in the expression of PD-1 [37]. However, as shown in this study, this downregulation is not achieved via the inhibition of mTORCs. Whether the inhibition of GSK3β can also downregulate Tim-3 expression in the exhausted CD8^+^ T cells remains to be elucidated.

IL-2-deprivation is one of the mechanisms through which the adaptive immune system is regulated [51,52,53,54]. This deprivation effect is often observed after several days, even though IL-2 starvation of cultured T cells can induce apoptosis within several hours [55]. The acute IL-2 starvation of our antigen-expanded CD8^+^ T cells lasted no longer than 24 h and was not associated with a notable decrease in viability of the cells prior to antigen rechallenge. However, without any obvious impact on cell viability, acute starvation had a dramatic and contrasting impact on the immediate and distal cytokine responses after antigen rechallenge. Specifically, the impact on the distal cytokine responses of antigen-re-expanded CD8^+^ T cells can indicate new avenues of intervention that could enhance these cells’ therapeutic performance.

Current immunotherapeutic approaches are attempting to enhance the performance of antigen-expanded T cells [56,57]. There are a variety of immunomodulatory approaches that can be applied, including genetic modifications [58,59] or pharmacological interventions in signaling pathways, namely, interventions in the GSK3β-mTORC signaling axis whose modulation was reported to impact the differentiation and effector responses of CD8^+^ T cells [37,46,60,61,62]. While genetic interventions are mostly irreversible, pharmacological approaches often need continuous effective concentrations of active compounds. Since GSK3β-mTORC signaling also regulates cell proliferation, the continuous presence of compounds that inhibit this axis also inhibits T cell proliferation [63,64,65]. In our study, the continuous presence of the active compounds was not indeed plausible because the GSK3β inhibitor TWS119 entirely abrogated re-expansion of the antigen rechallenged CD8^+^ T cells. However, acute conditioning with this inhibitor or the mTORC1 inhibitor rapamycin in the presence of IL-2 did not abrogate cell proliferation and, similar to acute IL-2 starvation, it enhanced the distal inflammatory responses of re-expanded CD8^+^ T cells. In contrast to rapamycin, the mTORC1/mTORC2 inhibitor Torin1 [42,66] showed no distal impact on re-expanded CD8^+^ T cells. This finding indicates that sustainable mTORC2 signaling is necessary for rapamycin (mTORC1 inhibition)- or TWS119 (GSK3β inhibition)-mediated enhancement of the distal inflammatory response of re-expanded CD8^+^ T cells. In this regard, acute GSK3β inhibition, prior to antigen rechallenge, can act as an enhancer of mTORC2 signaling [67], and acute mTORC1 inhibition with rapamycin or its disengagement due to acute IL-2 starvation can also elicit a compensatory mechanism leading to enhanced engagement of mTORC2 [68]. Regardless of how GSK3β-mTORCs are mutually regulated during acute conditioning with inhibitors and whether mTORC2 is indeed the key signaling player in this setting, acute conditioning of the GSK3β-mTORC signaling axis could represent a new avenue of intervention to enhance the functional performance of antigen-re-expanded CD8^+^ T cells. In this regard, future analyses of other markers of CD8^+^ T cell activation other than IFNγ and TNFα could further refine the understanding of how the GSK3β-mTORC signaling axis affects CD8^+^ T cell activation.

The approaches that rely on acute cell conditioning to modulate cell responses use pharmacological compounds whose long-term use would otherwise have nonspecific, toxic, or unwanted effects, such as abrogation of T cell proliferation [69]. These approaches often show that active compounds under acute or chronic conditions can deliver opposing effects [28,29] or even switch the quality of the cell effector response to the stimulus [27]. Acute conditioning of cytotoxic lymphocytes with a phosphatase inhibitor was reported to enhance the immediate cytotoxic response [70]. Our data also showed that acute conditioning of cytotoxic lymphocytes with IL-2 could enhance their immediate response. However, our data newly showed that this acute conditioning could also have a distal impact on the cells’ responsive phenotype after their re-expansion. These findings, therefore, indicate that acute modulation of cell signaling prior to cell activation may affect cell distal effector functions. As such, the compounds whose use has been so far limited due to their toxicities upon their chronic use might then be reconsidered for acute modulation of the cells to affect their distal effector functions.

## 4. Materials and Methods

### 4.1. Specimens

The source material was a buffy coat prepared from 450 mL of donated blood from healthy adult blood donor volunteers. The buffy coats were obtained from the Institute of Hematology and Blood Transfusion in Prague. Each donor provided signed written informed consent to participate in the study. All experimental protocols were approved by the ethical standards of the institutional and/or national research committee—the Ethics Committee of the University Hospital Motol in Prague (Project Identification Number 16-28135A; approval date: 25 June 2015) and performed in accordance with the 1964 Helsinki declaration and its later amendments or comparable ethical standards.

### 4.2. DC Preparation

The process is shown in Figure S1. Peripheral blood mononuclear cells (PBMCs) from buffy coats were isolated and fractioned into adherent and nonadherent cells as described previously [71], with the exception that Leucasep tubes (Greiner Bio-One, Frickenhausen, Germany) were used for PBMC isolation. The nonadherent fraction (lymphocytes) was cryopreserved. The adherent fraction (monocytes) was cultured at 37 °C and 5% CO_2_ in fetal bovine serum-containing culture medium [KM medium; RPMI 1640 medium (Thermo Scientific, Waltham, MA, USA), 10% fetal bovine serum (HyClone, GE Healthcare Life Sciences, South Logan, UT, USA), 100 U/mL penicillin-streptomycin, 2 mM GlutaMax (Thermo Scientific)] supplemented with GM-CSF and IL-4 (1000 IU/mL; Immunotools, Friesoythe, Germany) for 4 days. The cells were supplemented with GM-CSF and IL-4 (1000 IU/mL) and cultured for 1 day. The cells were harvested, pelleted at 240× *g* for 10 min at RT, and resuspended in fresh KM medium with GM-CSF and IL-4 (1000 IU/mL) at a concentration of 1 × 10^6^ cells/mL. The cells were supplemented with TLR3 agonist poly I:C (25 µg/mL; Hycult Biotech, Uden, The Netherland) and TLR7/8 agonist R848 (10 µg/mL; Enzo Life Sciences, Farmingdale, NY, USA), and 3 mL of the cell suspension was transferred to a flat-bottom 6-well plate and cultured (matured) for 18–24 h. The cells were harvested, γ-irradiated [32 Gy; Gammacell 3000 ELAN (Best Theratronics, Ottawa, ON, Canada)], and cryopreserved. For determination of DC maturation, the cells cultured in the presence (mDC; matured DC) or absence (iDC; immature DC) of poly I:C and R848 were transferred to a V-bottom 96-well plate (Nalgene, Rochester, NY, USA), pelleted, washed with ice-cold PBS containing 2 mM EDTA (PBS/EDTA), and stained with the following antibodies: CD11c-APC (Exbio, Carlsbad, CA, USA) in combination with CD80-FITC, CD86-PE, CD83-FITC (Beckman Coulter, Brea, CA, USA), or HLA-DR-Pe-Cy7 (Becton Dickinson, Franklin Lakes, NJ, USA) for 30–60 min at 4 °C. The cells were washed with ice-cold PBS/EDTA, supplemented with DAPI (100 ng/mL; Thermo Scientific), and immediately analyzed by a FACSAria II or BD LSRFortessa flow cytometer (Becton Dickinson). The obtained data were evaluated by FlowJo software (Tree Star, Ashland, OR, USA).

### 4.3. Enrichment and Expansion of Antigen-Specific CD8^+^ T Cells

The process is shown in Figure S2A. The cryopreserved buffy coat-isolated lymphocytes were reconstituted and cultured 18–24 h (37 °C, 5% CO_2_) in human plasma serum-containing culture medium [LM medium; RPMI 1640 medium, 5% human plasma serum (One Lambda, Canoga Park, CA, USA), 100 U/mL penicillin-streptomycin, 2 mM GlutaMax, 1 mM sodium pyruvate and nonessential amino acid mix (Thermo Scientific)]. The cells were pelleted at 240× *g* for 10 min at RT and resuspended in fresh LM medium at a concentration of 1 × 10^6^ cells/mL. The cell suspension (0.5 mL) was transferred to flat-bottom 48-well plates (Nalgene). Cryopreserved γ-irradiated DCs, prepared from two HLA-A2-incompatible allogeneic donors, were freshly reconstituted in LM medium at a concentration of 0.5 × 10^6^ cells/mL and mixed at a 1:1 ratio, and 0.2 mL of the DC mix was added to the wells of flat-bottom 48-well plates with 0.5 mL of lymphocytes (5:1 ratio of lymphocytes and DCs). The cells were resuspended and cultured (37 °C, 5% CO_2_) for 7 days. On day 5 of the DC-primed lymphocyte culture, the PC-3 prostate cancer cell line [72] was passaged (0.1 × 10^6^ of cells in 1 mL of KM medium) into a flat-bottom 48-well plate and then cultured (37 °C, 5% CO_2_) for 2 days. On day 7 of the DC-primed lymphocyte culture, the plate with the cultured PC-3 cells was cooled at 4 °C for 5–10 min and then UV-irradiated (312 nm, 2.55 J/cm^2^) with a Bio-Link BLX E312 crosslinker (Vilber Lourmat, Collégien, France) for 10 min at RT. The plate was cooled at 4 °C for 5–10 min and then again UV-irradiated as above. The plate was cooled at 4 °C for 5–10 min. All the supernatant was removed from the wells, and 0.75 mL of the 7-day-cultured allogeneic DC-primed lymphocytes, adjusted with LM medium to a concentration of 2 × 10^6^ cells/mL, was gently (dropwise) added. The PC-3-primed lymphocytes were then cultured (37 °C, 5% CO_2_) for 1 day. On day 8, the cells were gently (dropwise) supplemented with 0.5 mL of LM medium containing IL-2 (160 IU/mL; PeproTech, Rocky Hill, NJ, USA). The next day (day 9 of culture), the PC-3-primed lymphocytes were extensively resuspended, pooled, and transferred to a tissue culture flask (TPP, Trasadingen, Switzerland). The cell suspension was supplemented with an equivalent volume of LM medium with IL-2 (80 IU/mL). On day 11 of culture, the cell suspension was supplemented with one-third equivalent volume of LM medium with IL-2 (160 IU/mL). On day 12 of culture, the cell suspension was supplemented with an equivalent volume of LM medium with IL-2 (80 IU/mL). On day 14 of culture, the DC/PC-3/IL-2-expanded lymphocytes were cryopreserved.

### 4.4. Tim-3 Kinetics, Reversion Analysis, and Annexin V Assay

To determine the kinetics of the IL-2-mediated surface expression of Tim-3 in the 14-day DC/PC-3/IL-2-expanded lymphocytes, the cells were reconstituted in the absence of IL-2. After 18–24 h (37 °C, 5% CO_2_), the starved cells were washed and resuspended in fresh LM medium with IL-2 (80 IU/mL) and cultured for the indicated times. To determine the reversal of the cytokine-mediated surface expression of Tim-3 in the 14-day DC/PC-3/IL-2-expanded lymphocytes, the cells were reconstituted and cultured 18–24 h (37 °C, 5% CO_2_) in LM medium supplemented with IL-2 (133 IU/mL), IL-7 (13 IU/mL), IL-15 (133 IU/mL), IL-6 (666 IU/mL), or IL-33 (1333 IU/mL). The cells were harvested, pelleted, washed two times with LM medium, and then resuspended in LM medium supplemented, or not, with the corresponding cytokines. The cells were then cultured for 18–24 h. The cultured cells were transferred to a V-bottom 96-well plate (Nalgene), pelleted, washed with ice-cold PBS containing 2 mM EDTA (PBS/EDTA), and stained with the following antibodies: CD3-PerCP-Cy5.5, CD4-PE-Cy7 (eBiosciences, San Diego, CA, USA), CD8-Alexa Fluor 700 (Exbio), and Tim-3-PE (BioLegend, San Diego, CA, USA) for 30–60 min at 4 °C. The cells were washed with ice-cold PBS/EDTA, supplemented with DAPI (100 ng/mL; Thermo Scientific) and immediately analyzed by flow cytometry as described above.

Cell viability of DC/PC-3/IL-2-expanded lymphocytes after the cytokine starvation was determined by DAPI and annexin V staining. The cells were reconstituted and cultured (37 °C, 5% CO_2_) for 18–24 h in LM supplemented with IL-2 (80 IU/mL). The cells were pelleted, washed twice with LM medium, and resuspended in fresh LM medium with or without IL-2 (80 IU/mL), and cultured for 18–24 h. The cultured cells were transferred to a V-bottom 96-well plate (Nalgene), pelleted, washed with ice-cold annexin V binding buffer [73], and stained with annexin V-FITC (Exbio) for 15–30 min at 4 °C. The cells were rinsed with ice-cold annexin V binding buffer, supplemented with DAPI (100 ng/mL; Thermo Scientific), and immediately analyzed by flow cytometry as described above.

### 4.5. Cell Stimulation, Intracellular Cytokine Staining, and Cytokine Release

The expanded lymphocytes were reconstituted and cultured for 18–24 h (37 °C, 5% CO_2_) in LM supplemented with IL-2 (80 IU/mL). The cells were harvested, pelleted, and resuspended in the corresponding fresh medium at a concentration of 4 × 10^6^ cells/mL. Alternatively, the cells were supplemented with recombinant galectin-9 (20 μg/mL, R&D Systems, Minneapolis, MN, USA) 30 min before cell stimulation. Cultured PC-3 cells were rinsed with PBS, trypsinized, harvested, and resuspended in the corresponding LM medium at a concentration of 1 × 10^6^ cells/mL. The suspensions (100 µL) of lymphocytes and fresh PC-3 cells in the corresponding LM medium were combined in a U-bottom 96-well plate (Nalgene), extensively resuspended and cultured for 1 h (4:1 ratio of lymphocytes and PC-3 cells). The cells were gently supplemented with brefeldin A (BioLegend) and cultured for 4 h. The cells were transferred to V-bottom 96-well plates (Nalgene) and stained with live/dead fixable stain (Aqua, Waltham, MA, USA), fixed, and permeabilized as described [39]. The fixed and permeabilized cells were stained with the following antibodies: CD3-PerCP-Cy5.5, CD4-PE-Cy7 (eBiosciences), CD8-Alexa Fluor 700 (Exbio), TNFα-APC, and IFNγ-PE (Becton Dickinson) for 30–60 min at 4 °C. The stained cells were washed with PBS/EDTA and analyzed by flow cytometry as described above.

For determination of cytokine release, the expanded lymphocytes were stimulated as described above with the exception that the cells were not supplemented with brefeldin A and the cells were stimulated for 20 h. After the stimulation, 150 µL of supernatant was recovered from the U-bottom wells. The supernatant was cryopreserved or directly analyzed. The contents of TNFα and IFNγ released into the supernatant were determined as the concentration of cytokines released by 1 × 10^6^ cells/mL into the supernatant using Duo-Set ELISA (R&D Systems).

### 4.6. β-Catenin Translocation Analysis and Phospho Flow

The β-catenin translocation analysis was performed by imaging flow cytometry as previously described [39]. Briefly, DC/PC-3/IL-2-expanded lymphocytes were reconstituted and cultured (37 °C, 5% CO_2_) for 18–24 h with IL-2 (80 IU/mL). The cells were pelleted, resuspended in IL-2-containing medium (80 IU/mL) supplemented with TWS119 (7 µM), and cultured for 18–24 h. The cells were fixed and permeabilized as above and stained with CD3-PerCP-Cy5.5 (eBiosciences), CD8-Alexa Fluor 700 (Exbio), β-catenin-FITC (Miltenyi Biotec, Gladbach, Germany), and DRAQ5 (5 µM, Thermo Scientific) for 30–60 min at 4 °C. The stained cells were washed with PBS/EDTA and analyzed using ImageStreamX MKII (Amnis Corporation, Seattle, WA, USA), and nuclear localization of β-catenin was determined by a Pearson correlation using the IDEAS analysis software (Amnis Corporation). The proportions of cells with β-catenin translocated to the nucleus were evaluated.

Protein phosphorylation analysis was determined with phospho-specific flow cytometry. DC/PC-3/IL-2-expanded lymphocytes were reconstituted and cultured (37 °C, 5% CO_2_) for 18–24 h with IL-2 (80 IU/mL). The cells were pelleted, resuspended in IL-2-containing medium (80 IU/mL) supplemented with rapamycin (100 nM) or Torin 1 (100 nM), and cultured for 3 h. The cells were fixed, permeabilized, and stained with Alexa Fluor 488-labeled phospho-(Ser235|236) ribosomal protein S6-specific antibody (clone N7-548, Becton Dickinson) using the Cell Signaling Buffer Set A (Miltenyi Biotec, Gladbach, Germany) according to the manufacturer’s instructions. A fluorescent dye-labeled IgG antibody was used as a negative control.

### 4.7. Flow Cytometry Cytotoxic Assay

The 14-day DC/PC-3/IL-2-expanded lymphocytes were reconstituted and cultured (37 °C, 5% CO_2_) for 18–24 h in LM medium with or without IL-2 (80 IU/mL). The cells were pelleted, washed, and resuspended in fresh LM medium with or without IL-2 (80 IU/mL), and stimulated by coculturing with fluorescent TagFP635-PC-3 cells [39] at a ratio of 20:1 (lymphocytes:TagFP635-PC-3 cells). After 5-h coculturing, the cells were supplemented with Precision Count Beads (BioLegend), and the relative proportion of TagFP635-PC-3 cells to the Precision Count Beads determined by flow cytometry as described above. The cytotoxic activity was calculated as the difference between the relative proportion of TagFP635-PC-3 cells cocultured and not cocultured with DC/PC-3/IL-2-expanded lymphocytes.

### 4.8. Antigen Rechallenge and Re-Expansion of CD8^+^ T Cells

The 14-day DC/PC-3/IL-2-expanded lymphocytes were reconstituted and cultured for 18–24 h (37 °C, 5% CO_2_) in LM supplemented with IL-2 (80 IU/mL). The cells were pelleted, washed two times with LM medium, and resuspended in fresh LM medium with or without IL-2 (80 IU/mL). Alternatively, the cells were resuspended in IL-2-containing medium supplemented with 7 µM TWS119 (Sigma-Aldrich, St. Louis, MO, USA), 100 nM rapamycin, or 100 nM Torin1 [42] (Selleckchem, Munich, Germany). The cells were then cultured for 18–24 h. The cells were harvested, pelleted, and resuspended in the corresponding fresh medium at a concentration of 4 × 10^6^ cells/mL. Cultured PC-3 cells were UV-irradiated two times as above, rinsed with PBS, trypsinized, harvested, and resuspended in the corresponding LM medium at a concentration of 1 × 10^6^ cells/mL. The corresponding LM medium suspensions (100 µL) of lymphocytes and freshly irradiated/trypsinized PC-3 cells were combined in the U-bottom 96-well plate (Nalgene), extensively resuspended and cultured for 18–24 h (4:1 ratio of lymphocytes and PC-3 cells). The PC-3-reprimed lymphocytes were extensively resuspended and transferred to a flat-bottom 12- or 6-well plate, and all samples were supplemented with a double volume of fresh LM medium with IL-2 (4000 IU/mL) and cultured for 6 days. The cells were supplemented with an equivalent volume of LM medium with IL-2 (4000 IU/mL) every 2 days.

### 4.9. Statistical Analysis

The means and SEM values were calculated from the indicated sample size (*n*) using GraphPad Prism 6 (GraphPad software, La Jolla, CA, USA). Statistical significance (*p* < 0.05) was determined by the indicated test.

## 5. Conclusions

These data showed that acute conditioning of antigen-expanded CD8^+^ T cells before antigen rechallenge dictates their immediate and post-re-expansion functionality. Acute conditioning of the cells via the GSK3β-mTORC signaling axis before antigen rechallenge can enhance the cell functional performance after antigen-mediated re-expansion.

## Supplementary Materials

The following are available online at https://www.mdpi.com/2072-6694/12/12/3766/s1, Figure S1: Preparation of mature monocyte-derived DCs, Figure S2: Enrichment and expansion of antigen-specific CD8^+^ T cells, annexin V staining, β-catenin translocation, and phospho flow.
