# Supplementary material for: Acute Conditioning of Antigen-Expanded CD8+ T Cells via the GSK3β-mTORC Axis Differentially Dictates Their Immediate and Distal Responses after Antigen Rechallenge
Source: Cancers (Basel). 2020 Dec 14;12(12):3766. doi: 10.3390/cancers12123766 (PMC7765077; doi:10.3390/cancers12123766)
Supplement: Supplementary file 1 [file cancers-12-03766-s001.pdf]

cancers

Acute conditioning of antigen-expanded CD8+ T cells via the GSK3β-mTORC axis differentially dictates their immediate and distal responses after antigen rechallenge

Pavla Taborska, Dmitry Stakheev, Hana Svobodova, Zuzana Strizova, Jirina Bartunkova and Daniel Smrz

Supplementary Materials

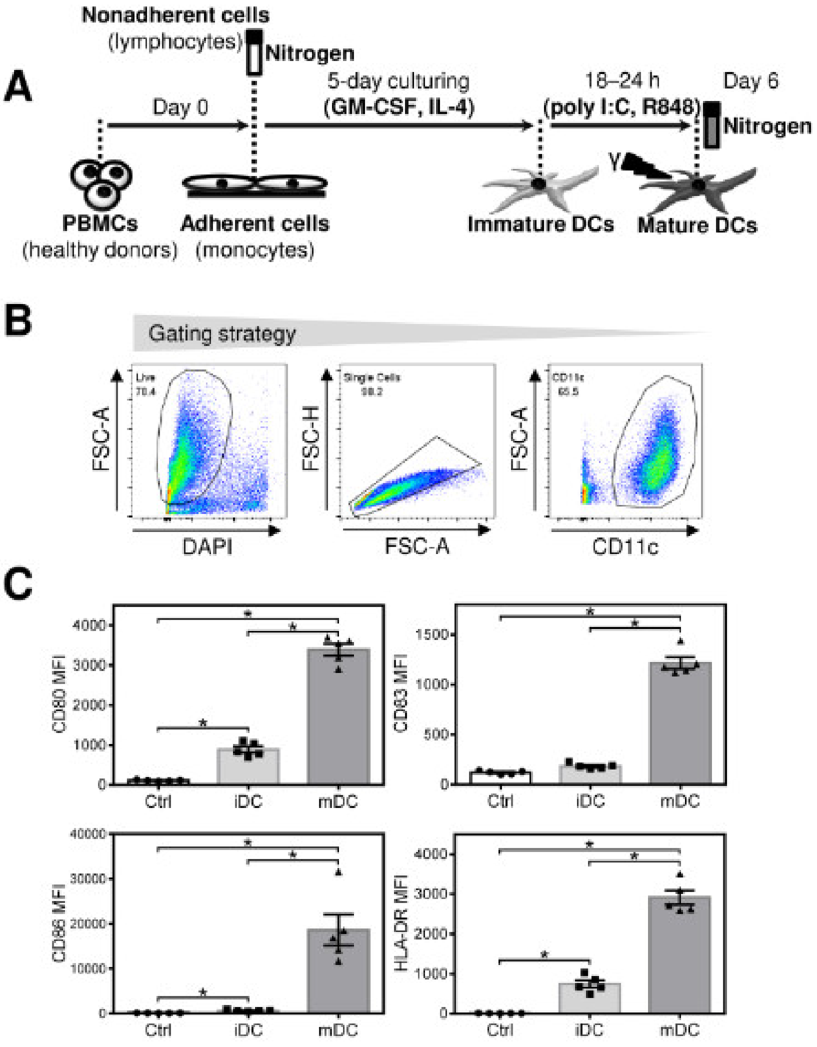

Figure 1. Preparation of mature monocyte-derived DCs. PBMCs from buffy coats were isolated and fractioned into adherent and nonadherent cells. The nonadherent fraction (lymphocytes) was cryopreserved and the adherent fraction (monocytes) was cultured for 4 days in the presence of GM-CSF and IL-4 (1,000 IU/ml). The cells were supplemented with fresh GM-CSF and IL-4 and cultured for 1 day. The cells were transferred to fresh medium with cytokines, and the concentration was

adjusted to 1 × 10^6 cells/ml. The culture was supplemented with poly I:C (25 µg/ml) and R848 (10 µg/ml), and the cells were allowed to mature for 18–24 h. The cells were harvested and γ-irradiated [32 Gy], and their maturation was determined by flow cytometry through the surface expression of DC maturation markers. (**A**) Schematic of DC preparation. (**B**) The gating strategy used to analyze flow cytometric data. (**C**) Intensities of the maturation marker staining (MFIs) of DCs ($CD11c^+$ cells) with no marker antibody used (Ctrl), cells not matured (iDC) or matured (mDC) with poly I:C/R848. In **C**, bars represent the mean of values determined in each group, and the significance of differences among the tested groups is indicated (*$P < 0.05$, $n = 5$ donors, 1-way ANOVA with the Tukey post-test).

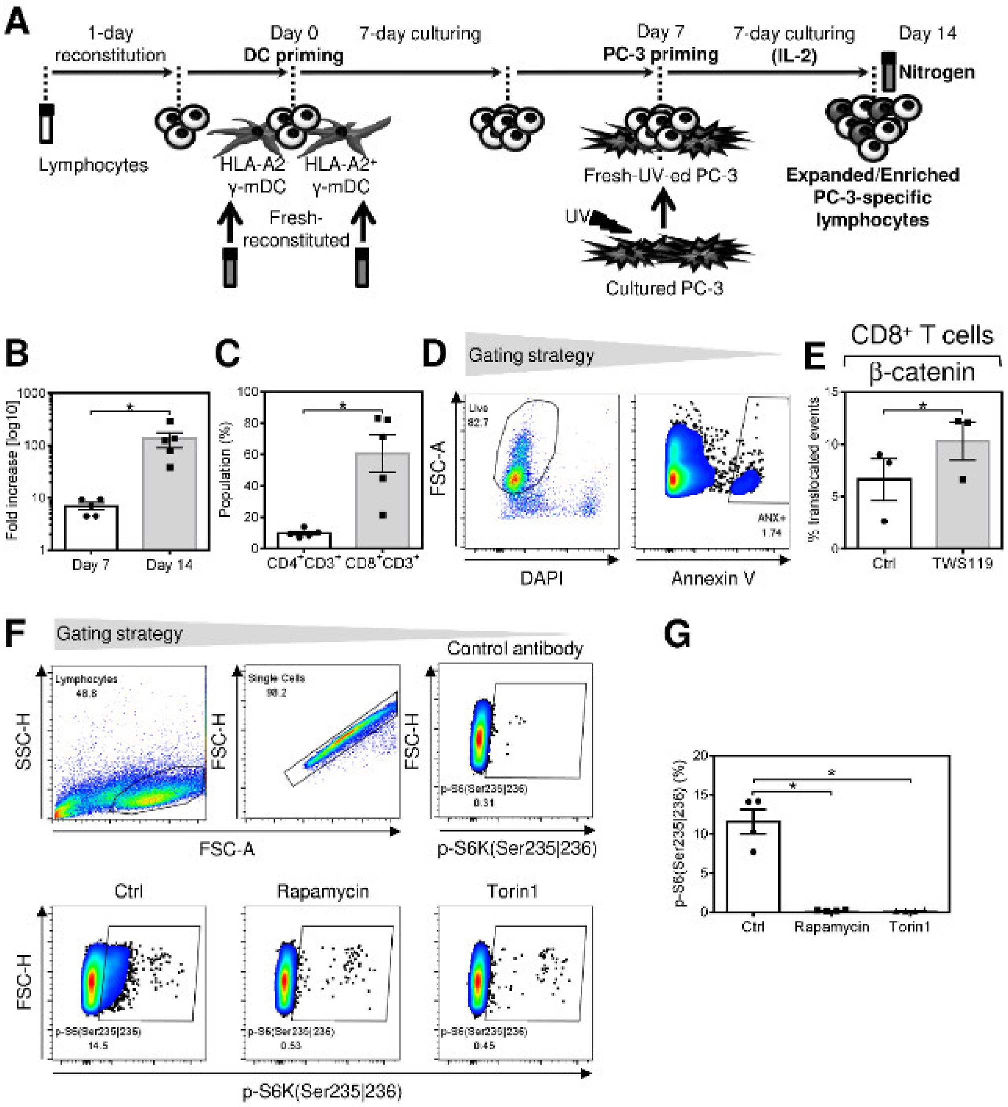

Figure 2. Enrichment and expansion of antigen-specific $CD8^+$ T cells, annexin V staining, β-catenin translocation, and phospho flow. (**A**) Schematic of enrichment and expansion of antigen-specific $CD8^+$ T cells. The cryopreserved buffy coat-isolated lymphocytes were reconstituted and cultured overnight. The cells were pelleted and resuspended in medium and cocultured in flat-bottom 48-well plates with a 1:1 ratio mix of γ-irradiated DCs from two HLA-A2-incompatible donors (5:1 ratio of lymphocytes and DCs) for 7 days. The DC-primed lymphocytes were then added to adherent UV-irradiated PC-3 cells in flat-bottom 48-well plates and cultured in the media with IL-2 for 7 days. On day 14 of culture, the DC/PC-3/IL2-expanded lymphocytes were cryopreserved. (**A**) Schematic of the enrichment and expansion of $CD8^+$ T cells. (**B**) Fold increase of the 14-day re-expanded lymphocytes on day 7 and day 14 of culture. (**C**) The proportions of $CD4^+$ and $CD8^+$ populations of T cells in the

14-day re-expanded culture. (**D**) The gating strategy used to analyze flow cytometry data of DAPI and annexin V stained cells. (**E**) Impact of TWS119 on β-catenin nuclear localization in the 14-day-expanded lymphocytes. The 14-day-expanded lymphocytes were reconstituted and cultured overnight (18–24 h) with IL-2 (80 IU/ml). The cells were pelleted, resuspended in IL-2-containing medium (80 IU/ml) supplemented with TWS119 (7 μM), and cultured for 18–24 h. The β-catenin nuclear localization in $CD8^+$ T cells ($CD8^+CD3^+$ cells) was determined by an imaging flow cytometer (ImageStreamX MKII) as the proportion of cells with β-catenin translocated to the nucleus. (**F**–**G**) Impact of rapamycin and Torin 1 on Ser235|236 phosphorylation of ribosomal protein S6 in the 14-day-expanded lymphocytes. The 14-day-expanded lymphocytes were reconstituted and cultured overnight (18–24 h) with IL-2 (80 IU/ml). The cells were pelleted, resuspended in IL-2-containing medium (80 IU/ml) supplemented with 100 nM rapamycin or 100 nM Torin1, and cultured for 3 h. The proportion of the phospho-Ser235|236 ribosomal protein $S6^+$ lymphocytes (p-S6K(Ser235|236) was determined by flow cytometry. (**F**) The gating strategy used to analyze flow cytometry data of phospho-Ser235|236 ribosomal protein S6-stained lymphocytes. A fluorescent-labeled IgG antibody was used as a negative control. (**G**) The evaluated proportions of the phospho-Ser235|236 ribosomal protein $S6^+$ lymphocytes (p-S6(Ser235|236)). In **B**–**C**, **E,** and **G**, bars represent the mean of values determined in each group. In **B**–**C**, and **E**, the significance of differences between the groups is indicated (*$P < 0.05$, **B**–**C**: $n = 5$ donors, **E**: $n = 3$ donors, paired 2-tailed Student's t test). In **G**, the significance of differences among the groups is indicated (*$P < 0.05$, $n = 4$ donors, 1-way ANOVA with the Tukey post-test).

Publisher's Note: MDPI stays neutral with regard to jurisdictional claims in published maps and institutional affiliations.

© 2020 by the author. Licensee MDPI, Basel, Switzerland. This article is an open access article distributed under the terms and conditions of the Creative Commons Attribution (CC BY) license (http://creativecommons.org/licenses/by/4.0/).
